# SARS-CoV-2 Infection Causes Relapse of Kleine-Levin Syndrome: Case Report and Review of Literature

**DOI:** 10.3390/neurolint13030033

**Published:** 2021-07-20

**Authors:** Marino Marčić, Ljiljana Marčić, Barbara Marčić

**Affiliations:** 1 Department of Neurology, University Hospital Center Split, Spinčićeva1, 21000 Split, Croatia; 2 Department of Radiology, Polyclinic Medikol, 21000 Split, Croatia; lmarcic@mefst.hr; 3 Department of Histology, School of Medicine, University of Mostar, 88000 Mostar, Bosnia and Herzegovina; barbara.marcic@mef.sum.ba

**Keywords:** SARS-CoV-2, Kleine-Levin, hypersomnia, hyperphagia

## Abstract

Recurrent episodes of hypersomnia, hypersexuality, compulsive eating, behavioral and cognitive disturbances, are the basic clinical features of Kleine-Levin syndrome (KLS). Our case report describes a patient who was diagnosed with KLS at the age of 20. With appropriate therapy, the disease had a satisfactory course until patient had a moderate form of SARS-CoV-2 infection, which led to a significant exacerbation of all symptoms. SARS-CoV-2 virus can cause almost any neurological disease, and relapse of KLS is another evidence of neurotropicity of the virus.

## 1. Introduction

Although Kleine-Levin syndrome was first described in 1925, although the clinical criteria for diagnosis are clear today, the disease itself, in terms of etiology, pathogenesis and treatment, remains a significant mystery [1,2]. Viral causative factors have been suggested because a significant number of patients with KLS before the onset of the disease had flu-like symptoms or an upper respiratory tract infection [3]. The SARS-CoV-2 virus is a single stranded RNA virus which primarily causes respiratory symptoms, but due to its pronounced neurotropism it can cause many neurological disease [4]. As described in his study by Mignot et al., the basic characteristics of the otherwise very rare Kleine-Levin syndrome are recurrent episodes of hypersomnia, compulsive eating, hypersexuality, behavioral and cognitive disturbances [5] KLS predominantly affects adolescent males, it is an exceptionally rare disease (affecting 1–5 person per million) [6], and it was first classified by the International Classification of Sleep Disorders (ICSD) in 1990. Today diagnosis of KLS is based on the criteria of ICSD-3rd edition from 2005.year [7] (Table 1) and is also known as “sleeping beauty syndrome”. The KLS has been classified into primary and secondary depending on identifiable underlying organic causes for recurrent symptoms. In our case report, we describe the exacerbation of Kleine-Levin syndrome after moderate SARS-CoV-2 infection, a combination that has not been reported previously.

## 2. Case Report

Our case report represents a young man who, at the age of 18, had excessive duration of sleep, an excessive daytime sleepiness, increased appetite, loss of interest in social activities during attendance of high school. He also had occasional visual hallucinations. The problems started after coming to high school. Occasionally he would fall asleep during class, on some days he would sleep more than 20 h per day, and in the waking state he had lots of time in a dreamy state. His appetite increased significantly and he began to eat compulsively and gained weight. He lost interest in sports and social activities since then. There were no sleep paralysis, fluid tests did not show any abnormality, brain magnetic resonance imaging (MRI) was noncontributory, Dopamine transporters scan of the brain (DaTSCAN) was normal and EEG shows α rhythm with normal reaction on eye opening and no pathological phenomena. IQ assessment was excellent, as a polysomnography followed by a multiple sleep latency test (MSLT). According to the ICSD-3 criteria, he was diagnosed with Kleine-Levin syndrome.

We gave him modafinil therapy in total daily dosage of 200 mg once a day. We also introduced risperidone therapy and after 15 months of therapy his total amount of sleep decreased to 10 h per day, his eating habits were normal and he has no hallucination. We gradually discontinued his modafinil and risperidone therapy so after 18 months of continuous therapy, he stopped taking all the recommended medications. Then he slept 8 h per day, there were no more eating disorders or hallucinations, he lost significantly on body weight. He continued his schooling properly, and the social component of life returned to normal. He regularly came for ambulatory checkups every 4 months. In the meantime, he finished college and got a job. He functioned properly in all segments of life: from sleep and eating hygiene to orderly mood and social contacts. For more than two years he was without any need for therapy. He even meanwhile, started a family and became a father.

**Figure 1:**
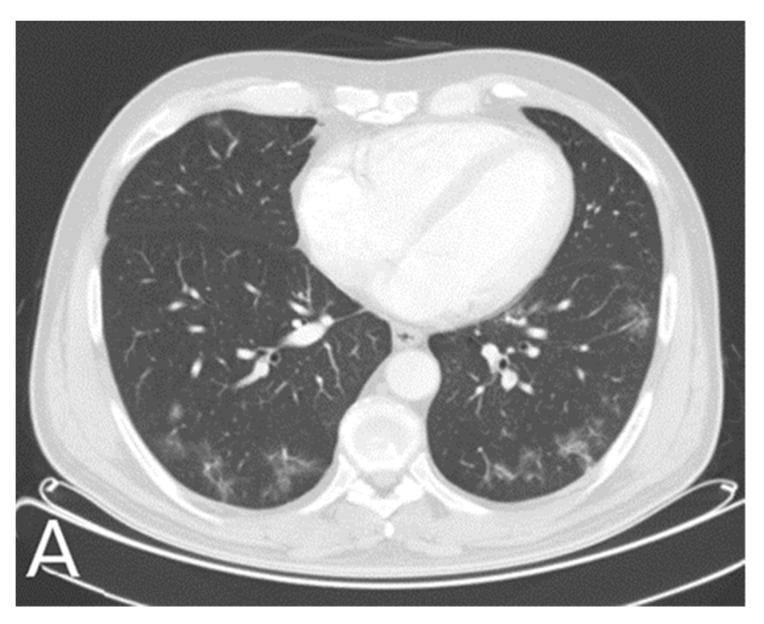
In January 2021, a 24-year-old male patient with history of Kleine-Levin disease was admitted to the emergency department as a SARS-Cov-2 positive with high fever, cough, dyspnea, anosmia, dysgeusia and fatigue. Symptoms worsened 6 days after the onset of infection, and lung MSCT showed areas of enhanced attenuation by the type of ground glass and patchy consolidations are seen on both sides-bilateral pneumonia (**A**).

In January 2021 year, due to respiratory symptoms and loss of sense of smell and taste, he reported to a family doctor and was diagnosed with SARS-CoV2 virus infection by reverse real time PCR test. In addition to moderate respiratory symptoms, nonspecific neurological symptoms such as headache, fatigue, dizziness have occurred. He was treated with antibiotic therapy, but he developed bilateral pneumonia (Figure 1). He was hospitalized for 10 days during which he was treated with antibiotic therapy, corticosteroid therapy and oxygen replacement therapy. 

**Figure 2:**
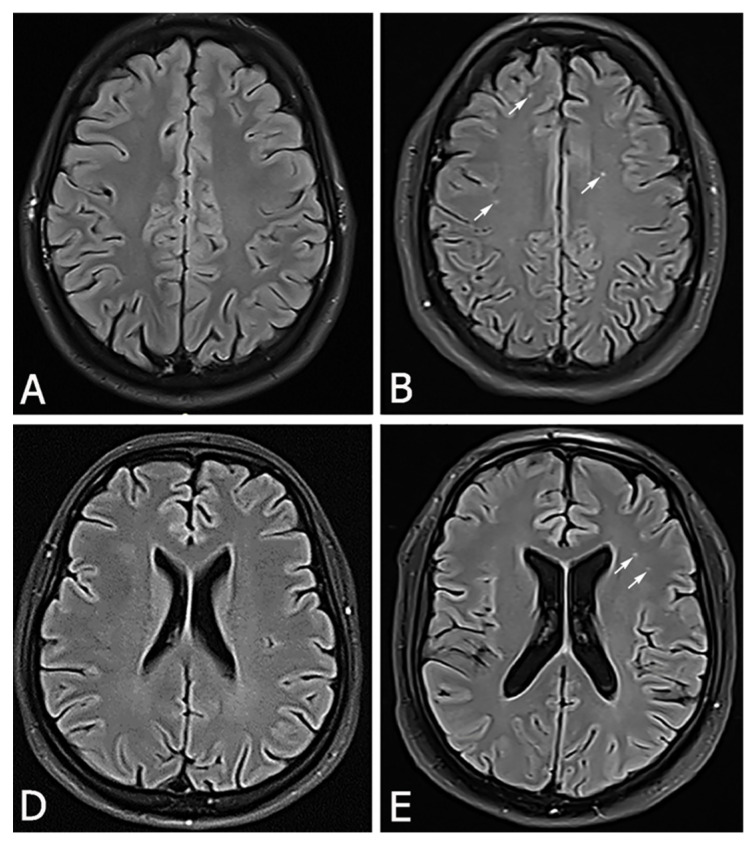
In September 2019.year, our patient had brain MRI (**A**,**D**) on which there were no pathological lesions (**A**,**D**). In March 2021.year, now 24-year-old same patient, 61 days after onset of moderate SARS-CoV-2 infection, had brain MRI who showed a hyperintensive lesions located bilaterally frontally subcortically and periventricularly on the FLAIR sequence (**B**,**E**).

One month after the onset of the infection, sleep and overeating occurred as it was in 2017. He slept again for more than 18 hours per day, in a wake state he felt constant fatigue and lethargy, he had increased appetite and overeating resulted in a significant increase in body weight. In addition, he lost interest in work and family, completely withdrawing into himself without interest in the environment with occasional outbursts of uncontrolled anger. His father and wife brought him in again for an examination. Apart from somnolence and disinterest, neurological status was normal, and brain MRI showed numerous changes in white matter (Figure 2B,E). EEG was completely normal. 

After discharge from the hospital, we also performed Transcranial Color Doppler (TCCD) on our patient with a breath holding test (BHT) which indicated decreased breath holding index (BHI) and impaired cerebral vasoreactivity (Table 2), as often occurs in patients after SARS-CoV-2 infection.

Modafinil was reintroduced into the therapy (200 mg), this time with lithium as a mood stabilizer (300 mg). A few months after the reintroduction of therapies, the patient has no more outbursts of anger, sleeps about 10 hours per day but in the waking state he feels sleepy. In addition to sleep disorders, there is also an eating disorder in the form of overeating and increased body weight. He is unable to go to work and does not function in the family either. He regularly comes for monthly checkups, takes therapy regularly, he had no side effects of the therapy and we have yet to see how long this time it will take for the symptoms to subside with proper therapy.

## 3. Discussion

In our case report, we presented a patient who had already been diagnosed for Klein Levin syndrome as a teenager. Exhaustive and comprehensive neurological workout did not find the cause of his disease, as in many patients with this syndrome, the cause has not been found. During the diagnostic workout, we paid great attention to the hypothalamus as the basic center in the control of sleep, appetite and sexual behavior [8]. Although no pathology were found, our patient was well with symptomatic therapy, we very successfully controlled his key symptoms and after a certain period of time therapy was gradually discontinued. Evaluation of the cerebrospinal fluid (CSF) and serological inflammatory markers are un-remarkable, electroencephalography (EEG) shows slowing in most patients during episodes without epileptic activity [9], but in our patient there were no pathological changes on the EEG. Single photon emission CT scanning during patients’ symptomatic periods can demonstrate hypoperfusion in the thalamus, hypothalamus, temporal lobes, orbito-frontal and parasagittal frontal lobes, and basal ganglia [10], but we were unable to do that test after our patient overcame a COVID infection. Fluorodeoxyglucose-positron emission tomography (FDG-PET) scanning in some patients may indicate asymmetric hypometabolism in the thalamus and hypothalamus [11,12], but not in our patient. Viral causative factors have been suggested, on the basis of the frequent report of flu-like symptoms at onset, and the most frequent precipitating factor is preceding infection [13]. The disease itself most often occurs in the colder periods of the year, especially in autumn and winter, when the number of respiratory infections is increased in the general population. The most common predisposing factors, in addition to those infectious, are psychological and physical stress, alcohol use or use of illicit drugs (marijuana, cocaine), sleep deprivation, physical exertion, traveling and head trauma. We did not find any predisposing factor in our patient at the time the disease was first diagnosed, but also at the most recent relapse of the disease. Only moderate SARS-CoV-2 infection coincided with the new relapse of his symptoms. The SARS-CoV-2 virus is a single stranded RNA virus which primarily causes respiratory symptoms, but an increasing number of patients also have neurological symptoms or neurological consequences of overcoming infection [14]. There is almost no neurological symptom that has not been described in the literature as a possible consequence of SARS-CoV-2 infection. Many COVID patients experienced non-specific neurological complications such as headache, dizziness, ageusia (loss of taste), anosmia (loss of smell), myalgia and fatigue. In moderate or severe forms of the COVID disease, can occur serious neurological symptoms such as prolonged headache, disturbance in consciousness, acute cerebrovascular disease (ischemic stroke, cerebral or subarachnoid hamorrhage), acute encephalopathy, encephalitis or meningitis, polyneuropathy, multiple sclerosis spectrum of disease and seizures. Direct viral invasion of the neural and endothelial cell by ACE2 receptors, diffuse endothelial inflammation, which impair cerebral vasoreactivity [15], coagulopathy and hyperactivity of the host immune system are the mechanisms by which the virus causes neurological dysfunction. The pathophysiological mechanism by which SARS-CoV-2 virus can trigger Klein Levin syndrome is unclear. It is probably a disorder at the level of the hypothalamus, either a direct invasion of neural and glial cells, or damage to the endothelial cells of the blood vessels essential for the perfusion of the hypothalamus. The way in which the SARS-CoV2 virus causes changes in the brain is most commonly associated with changes in the endothelium of the small blood vessels of the brain. The endothelium of small blood vessels has a large expression of ACE2 receptors which form an excellent channel for virus entry. Changes in the endothelium lead to diffuse endothelium damage which in turn causes changes in cerebral circulation. The new changes on the brain MRI in our patient are nothing but microcirculatory scars as a consequence of hypoperfusion, they are of vascular etiology, not demyelinating. Impaired vasoreactivity that we detected on TCCD in our patient also confirms that his cerebral endothelium is diffusely damaged after infection, but only some vascular lesion we have noticed on brain MR. Hypothalamic hypoperfusion may be the trigger for a new relapse of the disease. The most common findings on MR brain images after COVID infection were hyperintensive nonconfluent white matter lesions in the FLAIR sequence with postcontrast imbibition (such lesions are seen on MRI of the brain in our patient), hemorrhagic lesions, and diffuse microbleeds within the white matter in the SWI sequence in the medial temporal lobe area. Laminar cortical lesions are also common, which may indicate a disorder of vasomotor reactivity, but also numerous periventricular and juxtacortical hypertensive lesions in the FLAIR sequence with in-creased signal intensity on the DWI map or in the area of the corpus callosum [14]. The symptoms of Kleine-Levin syndrome are characterized by their intermittent and periodic nature [16]. The episodes themselves usually last between 1 and 3 weeks and cycle length can last from 2 to 4 months. Recurrences vary in frequency and in patients with adult onset, the disorder is less likely to resolve. As the disorder progresses and the patient ages, the cycle length increases. Symptoms tend to wane in severity as the disorder progresses and eventually spontaneously resolve in patients with adolescent onset [17]. Viral infections can be a common cause of exacerbations. The classic triad of hypersomnia, hyperphagia, and hypersexuality is not always present, and like our patient, they often exhibit some form of cognitive or mood impairment. As a rule, exacerbations of Klein Levin’s syndrome symptoms often present with the same pattern, so each new relapse of the disease can have identical clinical picture as the previous one. Our patient had the same clinical expression of the disease at the time he was diagnosed with the disease as well after SARS-CoV-2 infection.

Hypersomnia is an essential clinical symptom of KLS, and mandatory for diagnosis [18]. Usual sleep duration during episodes ranged from 12 to 24 h per day (our patient slept 16 h per day). Sleep symptoms changed from frank hypersomnia during the first episodes to a heavy fatigue accompanied by states between sleep and drowsiness. Patients tend to eat compulsively and in large amounts (megaphagia) with a preference for sweets and atypical food choices [19]. Hypersexuality is seen more frequently in boys than in girls and takes the form of increased sexual drive, frequent masturbation, sexual comments and unwanted sexual advances. Patients can be sexualy aggressive and had sexually charged language. Of all the key symptoms needed to diagnose Klein Levin syndrome, our patient had no symptoms of hypersexuality. Patients often withdraw from social interaction, and transient depression and anxiety can occur [20], as seen in our patient (Table 3).

The differential diagnosis of Kleine-Levin syndrome is extremely broad, basically the diagnosis is made by excluding all similar syndromes and very often the diagnosis is delayed by up to 4 years (Table 4).

Therapy for KLS must be supportive and medicaments [21]. We must allow patients to rest at home under supervision in a safe and comfortable environment, postpone or adjust school and work activities. The patient are not allowed to unattended operate a car or heavy machinery. We must monitor for symptoms of depression or anxiety, and between episodes, maintain consistent sleep-wake schedules, avoid alcohol and drug abuse. In the early stages of an episode we can consider amantadine or modafinil for excessive daytime sleepiness [22]. We can consider a short-term course of an antidepressant, mood stabilizer, anxiolytic or antipsychotic for psychiatric symptoms [23,24].

## 4. Conclusions

Although the true cause of Kleine-Levin disease is unknown, its increased incidence in the winter months and frequent previous viral infections have prompted thinking that its cause or trigger may be a viral disease. The causative agent was not found, but the SARS-CoV-2 virus in some as yet unexplained way activated a quiet disease in the presented patient. We showed new brain tissue lesions to the patient brain MRI scans and impaired cerebral vasoreactivity by TCCD and breath holding test. This is another proof of the pronounced neurotropicity of the SARS-CoV-2 virus and neurologists will obviously have a lot to do in the future with neurological consequences of this pandemic. In the future, we need well-managed registries of patients with SARS-CoV-2 infection and randomized studies with more subjects.

## Figures and Tables

**Table 1:** Diagnostic criteria for Kleine-Levin syndrome (ICSD-3).

A	at least two recurrent episodes of excessive sleepiness, each persisting for 2 days to 5 weeks,
B	episodes recur at least 1 per 18 months
C	normal alertness, cognitive function, behavior, and mood between episodes
In addition to the recurrent hypersomnia, the patient should also have at least one of the following:	
1. Hyperphagia	
2. Hypersexuality	
3. Abnormal behavior—irritability, aggression	
4. Cognitive abnormalities—ex confusion, derealization, hallucinations	
5. Hypersomnolence and related symptoms are not better explained by another sleep disorder, other medical, neurologic, or psychiatric disorder (especially bipolar disorder), or use of drugs or medications.	

**Table 2:** Transcranial Color Doppler (TCCD) and breath holding test (BHT) values.

	PSV (cm/s)	EDV (cm/s)	MV (cm/s)	PI	RI	BHTM (s)	BHI
rest	108	64	84	0.56	0.78		
after BHT	125	68	88	0.54	0.56	33	0.52

TCCD—transcranial color doppler, BHT—breath holding test, PSV—peak systolic velocity, EDV—end diastolic velocity, PI—pulsatility index, RI—resistance index, BHTM—breath holding time, BHI—breath holding index.

**Table 3:** Common symptoms in patients with Kleine-Levin syndrome (% of patients).

Hypersomnia	100%
Compulsive hyperphagia	80%
Disinhibition and hypersexuality	43%
Derealization	96%
Apathy	48%
Altered perception and temperature sensation	29%

**Table 4:** Differential diagnosis of Kleine-Levin syndrome.

	Clue for Diagnosis
Complex partial seizures	EEG
Lyme disease	Serology, brain MRI
Narcolepsy	Polysomnography
Herpes simplex virus encephalitis	CSF PCR test
Intermittent porphyria	Laboratory test
Klüver-Bucy syndrome (bilateral temporal lobe lesions)	Brain MRI
Metabolic encephalopathies	Laboratory test
Menstruation-related hypersomnia	History of symptoms temporal pattern
Sleep disordered breathing	History of OSA
Primary psychiatric illness (depression, bipolar disorder)	Hystory of psychiatric illness
Medication or substance abuse	History of drugs abuse

## Data Availability

The patient’s personal data are protected according to the GDRP regulations of the Republic of Croatia and the EU.
